# A Heat Emergency: Urban Heat Exposure and Access to Refuge in Richmond, VA

**DOI:** 10.1029/2023GH000985

**Published:** 2024-06-21

**Authors:** Peter Braun, Todd Lookingbill, Beth Zizzamia, Jeremy Hoffman, Jessica Rosner, Daisy Banta

**Affiliations:** ^1^ Department of Geography, Environment, and Sustainability University of Richmond Richmond VA USA; ^2^ Virginia Department of Health, Richmond and Henrico Health Districts Richmond VA USA; ^3^ Groundwork USA Yonkers NY USA; ^4^ L. Douglas Wilder School of Public and Governmental Affairs Virginia Commonwealth University Richmond VA USA; ^5^ Virginia Department of Health Office of Emergency Medical Services Glen Allen VA USA

**Keywords:** urban heat island, heat‐related health emergencies, built refuge, heat vulnerability, community planning

## Abstract

The urban heat island effect exacerbates independent climate change‐induced shifts toward longer, stronger, and more frequent heat extremes. Environmental inequity, driven by a history of racially motivated urban planning policies, has led particular demographics to bear the worst impacts of urban heat exposure and thus also climate change. These impacts cause adverse health outcomes in the form of heat emergencies. Through a novel demographic and spatial analysis of heat‐related illness Emergency Medical Services data from Richmond, Virginia, this study investigates the relationships between heat health emergencies and intra‐urban heat islands quantified through three heat exposure metrics. We also evaluate the accessibility of built refuge from urban heat in the form of public transit infrastructure, libraries, and government cooling centers in relation to these emergencies. We found that heat emergencies are inequitably distributed among racial, age, and socioeconomic groups in Richmond, particularly among residents identified as Male, Black or African American, 50+ years old, and experiencing mental health, intoxication, and/or homelessness. We found significant associations between the location of these heat emergencies and urban heat islands as estimated from remotely‐sensed surface and community science‐derived air temperature metrics, but not a co‐estimated heat index. We also found that available refuge facilities are insufficiently located to protect individuals with reduced mobility across areas with the highest number of heat‐related health emergencies. Community involvement in the mitigation and management of extreme heat threats, especially for those disproportionately impacted, is necessary to decrease the number of summertime heat illnesses.

## Key Points

- Heat‐related health emergencies are inequitably distributed in Richmond, Virginia across demographics
- Remotely‐sensed surface temperature, community science‐derived air temperature metrics are strong predictors of heat‐related health impacts
- Built refuge is essential for extreme heat management, inequitable access persists for low mobility and vulnerable residents

## 1 Introduction

Exposure to extreme heat can be deadly, especially for the most vulnerable populations within urban areas. Urban populations are growing; by 2030, it is estimated that 60% of the world's population will live in cities, potentially tripling the amount of urban land area since 2000 (Seto et al., 2012; United Nations, 2018). When temperatures rise globally due to human‐induced climate change, urban populations can experience even more intensified surface, air, and mean radiant temperatures than their rural and exurban neighbors due to the urban heat island (UHI) effect (Middel & Krayenhoff, 2019). The UHI effect is a phenomenon whereby metropolitan areas are significantly warmer than surrounding rural areas due to the human modification of land surfaces, mostly a lowering of surface albedo (Oke, 1982). Percent impervious surface area is a primary indicator of urban heat island formation and intensity due to the low albedo and high heat‐trapping capacity of the built surfaces in cities (Imhoff et al., 2010). The lack of evapotranspiration cooling from vegetation can compound the effect and make urban areas significantly hotter, especially during heat waves (Peng et al., 2012; Zhang et al., 2017).

The distribution of the UHI effect within cities is heterogeneous, as measured by on‐the‐ground surface temperature readings and remote sensing (Peng et al., 2012; Shandas et al., 2019). In a recent study of over 5,000 U.S. municipalities using Landsat based satellite imagery, McDonald et al. (2021) found that land surface temperatures in low‐income areas were on average 1.5°C warmer than those measured in high‐income areas and had 15.2% less tree canopy cover. This difference equates to an estimated 62 million fewer trees, affecting nearly 42 million people living in low‐income neighborhoods in those cities. In most cities, an intense surface UHI is measured in the urban core, with a gradient of lower temperatures radiating out to the periphery (Imhoff et al., 2010); however, these same areas may have lower near‐surface air temperatures due to shade canyons from building height variations (Shandas et al., 2019). Ziter et al. (2019) found city blocks with the coolest daytime temperatures had at least 40% tree canopy cover, though effects of canopy cover were less apparent at night and nighttime temperatures were more strongly correlated with percent impervious land cover. A study of the UHI in Madison, Wisconsin found seasonal variation in the intensity of UHI, with higher intensity in the warmer summer months and lower intensity in cooler winter months (Schatz & Kucharik, 2014). However, as the UHI effect may protect urban communities from cold related mortality in winter in other regions, absolute temperatures are of utmost concern for public health during the warm summer months (Macintyre et al., 2021). Recent work has also focused on modeling the variations of moist heat stress in urban areas, as the built environment lowers ambient humidity in more highly developed landscapes, potentially complicating the interpretation of urban heat metrics that do not include the effect of humidity on heat exposure (Chakraborty et al., 2022, 2023).

The ability of UHI to exacerbate and prolong elevated temperatures poses a serious threat to human health. Prolonged and acute exposure to high heat can cause heat‐related illnesses, such as heat stroke or heat exhaustion, and can trigger life‐threatening health emergencies such as heart attacks, as increased blood flow for physiological cooling puts stress on the cardiovascular system (Vaidyanathan et al., 2020). Approximately 65,000 Americans seek medical attention annually for heat‐related illnesses (CDC, 2016). In Virginia, days of “tropical‐level” heat and humidity (“very warm—typically associated with the location's highest temperatures—and humid, muggy, and uncomfortable, conditions that are prevalent in summer”) experience a 179% increase in heat‐related emergency department visits and a 217% increase in heat‐related hospitalizations (Woolf et al., 2023). Extrapolating these data nationally, extreme heat costs an estimated $1 billion in health care expenses every summer (Woolf et al., 2023). Estimates of average annual heat exposure mortality across the United States vary widely, with one study identifying 1,300 deaths annually in U.S. cities from 1975 to 2004 in excess of base mortality, and another estimating 12,000 deaths annually nationwide throughout the 2010s (Kalkstein et al., 2011; Shindell et al., 2020). Climate change is projected to increase heat‐related mortality across the United States by an additional 36,000 to 97,000 deaths annually, depending on the climate change scenarios used in models (Shindell et al., 2020). Pre‐existing health conditions, such as chronic respiratory and cardiovascular diseases, as well as drug use and some physical and mental disabilities, make a person physiologically more vulnerable to heat‐related illnesses (Vaidyanathan et al., 2020).

Most, if not all, heat‐related emergencies and deaths are considered avoidable by the Centers for Disease Control and Prevention (CDC), especially if the potentially exposed individuals have access to sufficient resources and shelter to reduce their exposure (Vaidyanathan et al., 2020). However, not all urban dwellers have access to these resources due to historic lack of private and government investment, often drawn on racial lines (Hoffman et al., 2020; Wilson, 2020). Communities on the front lines of heat exposure can be better protected, for example, by increasing urban tree canopy through the creation of new parks and street tree planting. A study of Washington, D.C. found that converting as little as 10% of roadways to grass and 50% tree shading can reduce average air temperatures by 4.1°C, with significantly greater decreases in temperatures on building faces (Loughner et al., 2012). Urban greening not only improves air quality and reduces direct heat exposure for people, but also reduces building temperatures, which has the effect of lowering energy usage and associated greenhouse gas emissions (Loughner et al., 2012; McDonald et al., 2020). Other mitigation strategies include improved heat‐health messaging and communication strategies, enhanced identification of and outreach to at‐risk populations, increased cooling centers, and decreased dark and impervious surfaces (Keith & Meerow, 2022; Randazza et al., 2023).

The risk from extreme heat is not equally distributed. Racist policy and planning decisions, from redlining and racial covenants to interstate highway construction and sacrifice zones, have created a physical and socioeconomic landscape of inequality with poorer communities of color, on average, experiencing significantly higher temperatures with less coping infrastructure than their wealthier, predominantly white counterparts (Benz & Burney, 2021; Hoffman et al., 2020; Hughes, 2011; Krimmel, 2018; Saverino et al., 2021). The association between health, wealth, and racial factors has also been well documented. For example, chronic respiratory and cardiovascular diseases disproportionately impact low‐income communities and communities of color (Gronlund, 2014). Low‐income individuals experiencing heat‐related illnesses may experience more severe health outcomes, as they are less likely to seek immediate medical treatment due to inaccessibility to healthcare or the threat of catastrophic medical bills and lost wages (Gronlund, 2014). Nationally, the most vulnerable demographics include men, individuals ages 65 years and older, and Native Americans (Vaidyanathan et al., 2020). When drawing connections between UHI and health outcomes, racial demographics and socioeconomic status must be considered as confounding variables (Fiscella & Williams, 2004).

While the connection between sociodemographic factors and exposure to extreme heat in cities has been well established, there is an emerging literature seeking to establish a direct connection between observed health outcomes and urban heat metrics (e.g., Li et al., 2022). The purpose of this study is to assess the associations between Emergency Medical Services (EMS) responses (e.g., ambulance responses to 9‐1‐1 calls) for heat‐related illnesses and three common urban heat intensity metrics in Richmond, Virginia. By disaggregating these emergencies across age, gender, racial demographics, and associated underlying risk factors, we hope to better inform future resource distribution, government investment, and community education in vulnerable areas of the city, while providing a case study on how different urban heat metrics can be evaluated alongside direct health outcome data. This study also aims to identify deficiencies in the quantity and spatial distribution of heat resilience resources like cooling center access in Virginia cities (Allen et al., 2022). We first quantify the spatial pattern of heat‐related EMS incidents and determine if these events are clustered, random, or evenly distributed among Richmond census block groups. We then compare this pattern to maps of heat distributions that we have previously created for the city (CAPA Strategies, 2021; Shandas et al., 2019). Our null hypothesis assumes there is no spatial relationship between any of the three metrics of UHI intensity (i.e., land surface temperature, air temperature, and heat index) and the number of heat‐related illness EMS responses in that same census block group. We then determine whether heat resilience resources, including cooling stations and public libraries, are effectively distributed to serve as refuge during heat events. Our second null hypothesis is that there is no difference in the accessibility of heat resilience resources throughout the study area.

## 2 Methods

### 2.1 Geographic Focus

The City of Richmond, Virginia is located at the falls of the James River on the east coast of the United States and has a population of 226,610 people, making it the fourth largest city in Virginia (Census, 2020). Prior to European colonization, the falls of the James River was the seat of the Powhatan Confederacy, an Algonquin‐speaking indigenous nation which stretched across much of the tidewater region of Virginia. Richmond was settled by English colonists in 1737 by Colonel William Byrd II and became the capital of the Virginia Colony in 1780. Richmond also served as the capital of the Confederacy for much of the U.S. Civil War. As the capital of the Commonwealth of Virginia today, Richmond is widely known for its vibrant arts scene, urban parks, and outdoor recreation, as well as its historic character.

### 2.2 Data

This study used (a) 2020 U.S. Census Data and American Community Survey 5‐Year Estimates on sociodemographic patterns for the city, (b) three different measures of the spatial distribution of heat throughout the city, (c) data from the City of Richmond on heat resilience infrastructure, and (d) data from the Virginia Department of Health Office of Emergency Medical Services on heat‐related illness EMS incidents.

We evaluated three different measures of heat distribution in Richmond. Each heat metric data set, though temporally limited, is representative of the primary heat season, primary heat‐sensitive weather type for the Richmond region (humid/warm Spatial Synoptic Classification) (Lee & Sheridan, 2018). This study tests the spatial structure of Richmond's UHI and not necessarily the individual intensity of a given day. The first was based on Landsat Collection 2 Level‐2 Land Surface Temperature (LST) data generated using the thermal infrared band (TIRS) from Landsat 8. Imagery of Richmond was acquired from 30 June 2021, 11:46 a.m. EDT and used to estimate temperature using standard Landsat surface temperature algorithms (Cook et al., 2014; USGS, 2021). Landsat‐based LST data represents the temperature of the Earth's surface (or skin temperature), which can differ from measures of air temperature experienced by people. Nevertheless, because these data are readily available, they are used in a variety of applications to study heat‐related issues in urban environments (Hsu et al., 2021; Li et al., 2013).

**Figure 1:**
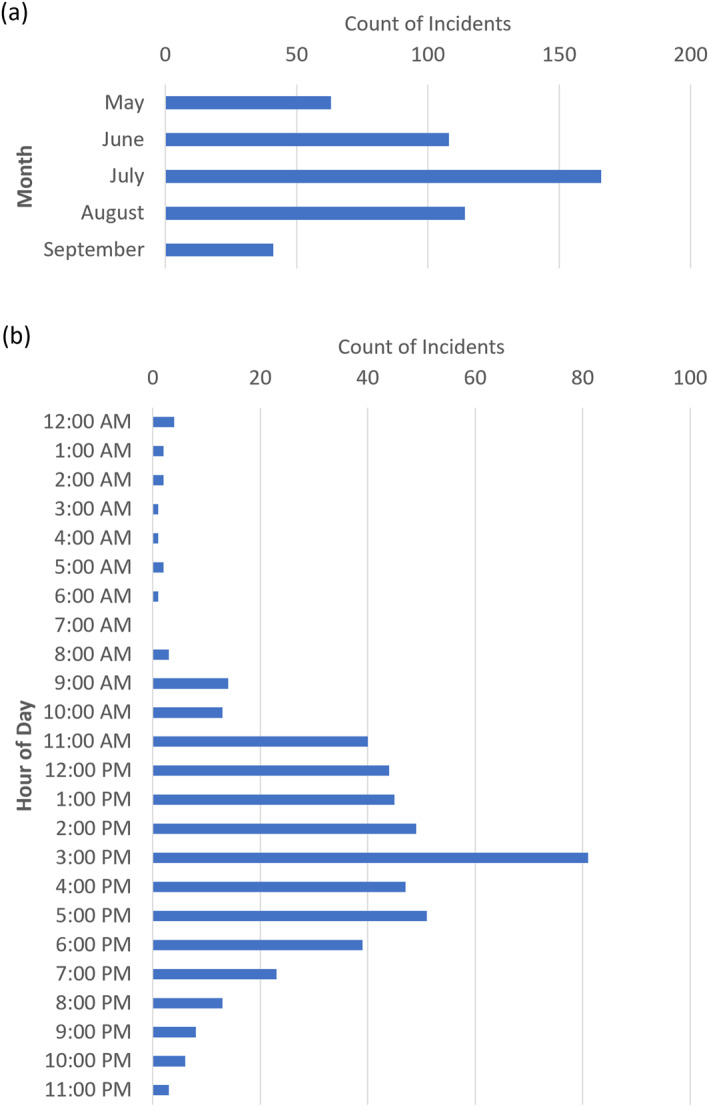
Count of heat‐related EMS incidents (*n* = 492) by (a) month and (b) hour of day (May–September 2016–2022; Richmond, VA).

A second representation of Richmond's heat distribution was generated from 15 July 2021 ground measurements of ∼2 m ambient air temperature (AT) following the methods of Shandas et al. (2019). From 3:00 to 4:00 p.m. on that afternoon, groups of community scientists familiar with the city drove automobiles on 12 separate, non‐intersecting routes to collect tens of thousands of georeferenced measurements using thermistors attached to their cars that recorded temperature at one‐second intervals following a well‐established community science methodology (Hoffman et al., 2022; Shandas et al., 2019). Air temperature measurements were then combined with Sentinel‐2 land cover data and run through a machine learning algorithm to create a predictive air temperature map for the entire city. Cross‐validation by holding out 30% of the data from the predictive modeling (Voelkel & Shandas, 2017) yielded an adjusted R‐squared value of 0.95 for this time period (CAPA Strategies, 2021), suggesting high levels of predictive power. Additionally, the date (15 July 2021) and time of measurements (3:00–4:00 p.m.) also correspond with the peak month of heat‐related EMS incidents (July) and peak hour of day for heat‐related EMS incidents (3:00–4:00 p.m.) in Richmond from 2016 to 2022 (Figure 1).

The third measure of Richmond's heat distribution combined the predictive model of air temperature generated from the 15 July 2021 data with direct measurements of relative humidity collected from those same sensors. Although less frequently considered in heat island research, atmospheric humidity may act as a valuable modifier on temperature in predicting heat stress in urban areas where developed land uses reduce atmospheric water vapor relative to areas with higher amounts of vegetation (Chakraborty et al., 2022). Because humans respond to heat by sweating, conditions of high heat and high humidity may be more dangerous to health than conditions of high temperature alone. To test the potential impact of high moisture‐related heat stress, we adjusted recorded temperatures for high and low relative humidity to create a heat index (HI) value following established methods (CAPA Strategies, 2021; NWS, 2022).

A data set of heat‐related EMS responses recorded in the Virginia Pre‐Hospital Information Bridge during the May‐September heat season from 2016 to 2022 was made available by the Virginia Department of Health (VDH) Office of Emergency Medical Services (OEMS) through a data use agreement. The *Code of Virginia* 32.1–116.1 requires every licensed EMS agency in the Commonwealth to report all EMS call records to this state data repository. EMS call records include both responses to 9‐1‐1 calls and to other EMS services provided to the public (e.g., ambulance standbys at sporting events or outdoor festivals). The case definition used to identify heat‐related illnesses was adapted by the study team for EMS data from the syndromic surveillance definition established by the Council for State and Territorial Epidemiologists (CSTE, 2016). EMS incidents were included in the data set if the reported Cause of Injury Code or Situation Complaint Statement was consistent with a heat‐related illness and the Scene Incident Postal Code was located in Richmond City, Virginia. As determined using *International Classification of Disease*, *Tenth Revision* (ICD‐10) codes for causes of injury, heat‐related illnesses in this data set included incidents where injuries were reported to be caused by dehydration, sunburn, or the effects of excessive natural heat and light (sunlight). Incidents were also included when the situation complaint statement referenced any variation of heat, heat exhaustion, heat cramp, heat‐stroke, sun stroke, hyperthermia, or overheating AND some combination of heat‐related symptoms, such as dehydration, electrolyte imbalance, sunburn, fatigue, stress, or heat‐risk situations, such as being in a car or being outside, or being found lying down. Information on the air temperature at each of the incident locations was also extracted by VDH OEMS, as described below. To protect the personal health information of patients, the spatial locations of incidents were aggregated and reported at the census block group level, with maps created at only the census tract level. Therefore, each heat‐related EMS incident record included the census block group in which the emergency response occurred.

The heat‐related EMS record data set also included elements that captured extensive information from the EMS response (e.g., patient racial/ethnic and age demographics, health history, cause of patient's injury or illness, geographic location of event, etc). It is important to note that information about patient risk factors was extracted from the Patient Narrative field in the EMS record, as no discrete data elements exist in which Virginia EMS clinicians can record information specific to heat‐related illness risk factors (e.g., the patient's activity prior to onset of illness). The Patient Narrative captures free text summaries of EMS/patient interactions and varies in detail from clinician to clinician. This method presents inherent limitations for identifying risk factors and is likely to result in an under‐reporting of health disparities. For example, homelessness and substance use may be underreported in the data set as there is no mandate to include this information. Furthermore, clinicians may be hesitant to include supplementary information in the narrative without substantive proof due to stigma and concerns their narratives may be questioned.

A Richmond City boundary polygon layer was obtained from the City of Richmond (Richmond GeoHub, 2020). Because no reported health emergencies were located within the James River, the Richmond City boundary was modified to remove this large water body from the study area. Point layers of city‐operated cooling facilities, Richmond Public library locations, Richmond community centers, and Greater Richmond Transit Company (GRTC) bus stop locations were also obtained from the Richmond GeoHub. For the purposes of this analysis, refuge is defined as city‐operated resources which are freely open to the public during a heat event. Richmond's public libraries are the foundation of the city's refuge system, as all nine library locations have been designated as cooling centers since 2020, with access limited to normal hours (Department of Social Services, 2020). A limited number of additional cooling center locations (1–3 locations each summer since 2009) are announced by social services via the City's website, social media, and local news outlets when air temperatures or heat indices reach or exceed 95° Fahrenheit.

### 2.3 Analyses

#### 2.3.1 Demographic Analysis

Heat‐related EMS incidents were disaggregated by demographics and compared to the city's overall demographic characteristics to assess whether common heat vulnerability factors are supported by Richmond's heat‐related EMS incident data. The data were filtered by conventional age brackets of 0–4, 5–19, 20–34, 35–49, 50–64, and 65 years and older. The heat‐related EMS incidents were also grouped by gender and the races and ethnicities reported in the EMS patient care data. The concepts of race and ethnicity are defined socially and culturally with the aim of identifying important cultural and social groups for statistical reporting and health improvement purposes. Races and ethnicities listed encompass all categories reported in the EMS heat‐related illness data set, but do not cover all races/ethnicities generally reported in census data sets. Two cases listed as “Other race” or “More than one race” were not included in this analysis.

Information on EMS patients' risk factors for experiencing heat‐related illnesses (including cognitive impairments, job risks, homelessness, and pregnancy) and activities engaged in prior to symptom onset were extracted from patient care report narratives. The proportions of cases experiencing specific risk factors and participating in each activity were calculated. Lastly, incident location types were categorized. Incident location types of “sidewalk,” “local residential or business street,” and “street or highway” were aggregated into the category “sidewalk or street.” Instances of “residence—single family home” and “residence—apartment or townhouse” were aggregated into “residence.” Locations of “recreation area—not otherwise specified,” “recreation area—public park,” “sport facility—field,” “sport facility—not otherwise listed,” and “wilderness area” were aggregated into the category “sport or recreation facility.” The percentage of cases in each incident location category was calculated.

#### 2.3.2 Heat Model Analysis

To determine if the locations of heat‐related EMS incidents were associated with or clustered near locations of elevated heat metrics, a random set of location points was created to act as a control group. The values of LST, AT, and HI were extracted from the underlying grid values of the three urban heat island rasters at the location of each heat‐related EMS incident and for each of the 492 randomly located points in the control group. The bilinear interpolation method was used to extract values, which calculates the value of each point by averaging the values of the surrounding four pixels. Bilinear interpolation was used because these locations are approximate and pixel size is small, while also controlling for outliers in the raster data. One‐tailed *t*‐tests for independent samples were performed to determine if the mean temperatures of the locations of heat‐related EMS incidents were greater than the mean temperature of the randomly selected points within the study area.

The three different measures of urban heat islands (LST, AT, HI) were also aggregated by census block groups to match the resolution at which the heat‐related EMS incident data could be reported. We evaluated the ability of each heat measure to differentiate high‐risk areas of the city by identifying the block groups with the top 20% (highest quintile) number of heat‐related EMS incidents during the study period. One‐tailed *t*‐tests were conducted to determine for each of the LST, AT, and HI data sets if their mean temperature values were higher for these block groups than for the block groups without any recorded heat‐related EMS incidents, which comprised the bottom 20% (lowest quintile) of the heat‐related EMS incident data. To protect patient confidentiality, all maps visualizing the spatial pattern of heat‐related EMS incidents provided in this study have been aggregated to the census tract level, following VDH data reporting policies.

#### 2.3.3 Heat Resilience Resource Analysis

Access to safe heat resilience resources during a heat event was assessed in this study. Utilizing data available from the City of Richmond Office of Sustainability's Climate Equity Index, we imported the locations of the libraries and three emergency cooling centers located in the city.

To determine the distance between heat‐related EMS incidents and refuge locations, walkshed buffers surrounding the refuge locations (“refuge zones”) were created. The count of heat‐related illness incidents within a walkshed was used as an estimate of the community's need for the refuge location. To determine accessibility, we assumed that exposed individuals did not have access to a car or faster method of transport and, therefore, were walking to seek refuge. In a similar study, acceptable walking distance was determined by a 15‐min radius and average walking speeds for “sedentary elderly (1.4 km/hr), average elderly (3.5 km/hr), and active adults (5.6 km/hr)” (Voelkel et al., 2018). These walking rates resulted in three distances of 0.35 km, 0.875 km, and 1.4 km for slow, normal, and fast walkers, respectively. Using latitude and longitude of heat‐related illness incident locations, the number of incidents that fell within each refuge zone was calculated, first for the libraries alone, then for the combined refuge zones of libraries and the three additional cooling centers.

Potential improvement in refuge zone coverage was evaluated by considering how the network could be enhanced with additional cooling centers. There are 24 city‐operated community centers in Richmond which could be used as refuge during a major heat event; however, they are not officially listed by the city for this purpose. Walkshed buffers of 0.35, 0.875, and 1.4 km around these centers were created to represent potential refuge zones. The number of heat‐related EMS incidents in Richmond that occurred within the combined refuge zones of libraries, cooling centers, and community centers was calculated. Community centers were then ranked by the number of heat‐related EMS incidents within each walkshed to determine the potential impact of these community centers as additional refuge facilities.

#### 2.3.4 Bus Stop Analysis

To determine if bus stop locations were associated with locations of high heat, we used a method similar to that used to assess temperature at locations of heat‐related EMS incidents. We calculated the mean AT for all bus stops in the city and compared that value to the mean AT for an equivalent number of randomly distributed control points located within three m of a main artery and secondary roads in the city. A two‐tailed *t*‐test for independent samples was used to determine if there was a significant difference between the mean temperature of bus stop locations and the randomly selected locations along Richmond's road network.

Further, to determine if heat‐related EMS incidents occurred in close proximity to bus stops, we recorded the distance between the latitudinal and longitudinal coordinates of each incident and the closest bus stop. We then calculated the distance between each incident and the closest of our randomly distributed bus stop control points. We compared the cumulative frequency distributions of these two sets of distance values (observed vs. expected if bus stops were no different than random), and any difference between the two distributions was evaluated using a two‐tailed Kolmogorov‐Smirnoff test.

The software used to analyze and visualize the data included ArcGIS Pro 3.1, Microsoft Office Excel, and R 4.1.2.

## 3 Results

### 3.1 Demographic Analysis

**Figure 2:**
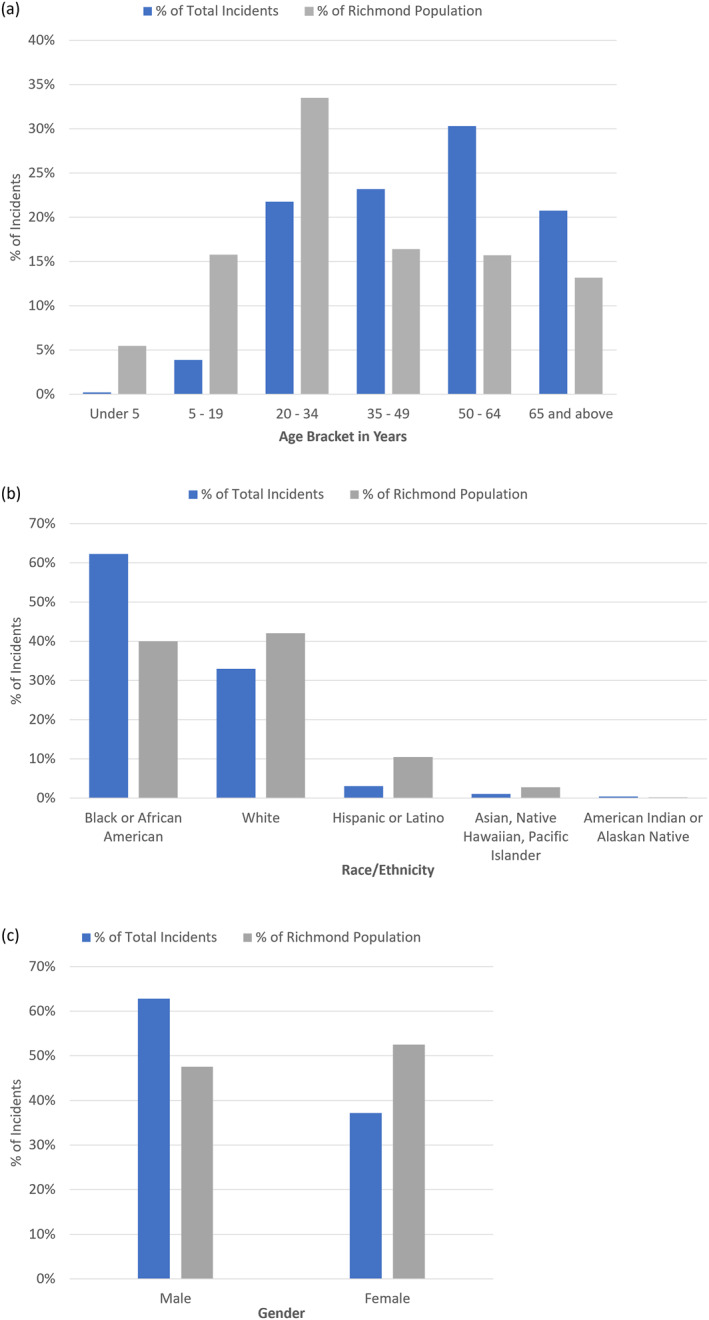
Percent of heat‐related illness EMS incidents (May–September 2016–2022; Richmond, VA) and percent of Richmond population by: (a) age bracket, (b) race or ethnicity, and (c) gender.

A total of 492 heat‐related EMS incidents occurred in the City of Richmond from 2016 to 2022. Individuals under 5 years of age and between the ages of 5–19 years made up the lowest number of these incidents (*n* = 20), accounting for 0.2% and 3.9% of the total burden, respectively (Figure 2a). Individuals between the ages of 50 and 64 years constituted the age group experiencing the largest proportion of heat‐related illnesses (30.3%, *n* = 149), while 35‐ to 49‐year‐olds and 20‐ to 34‐year‐olds experienced 23.2% (*n* = 114) and 21.7% (*n* = 107) of heat‐related illnesses, respectively. Individuals ages 65 years and above constituted 20.7% (*n* = 102) of cases. Patients in the oldest three age brackets represented a disproportionately larger number of heat‐related EMS incidents compared to Richmond's overall age demographics, while patients in each of the three age brackets under the age of 35 years represented a disproportionately smaller proportion of cases compared to the general population (Census 2020; Figure 2a).

Black or African American patients made up a disproportionately high percentage of total heat‐related EMS incidents during the study period compared to the Black or African American population of Richmond, representing 62.2% (*n* = 306) of the health burden but only 39.9% of the wider Richmond population (2020 Census; Figure 2b). The opposite relationship was found for white patients, who represented 32.9% (*n* = 162) of total heat‐related EMS incidents while making up 42.0% of the Richmond population. Hispanic or Latino residents accounted for only 3.0% (*n* = 15) of cases while representing 10.5% of the Richmond population. Patients documented as male represented a disproportionately large percent (62.8%, *n* = 309) of heat‐related EMS incidents during the study period compared to female patients (37.2%, *n* = 183) (Figure 2c).

**Figure 3:**
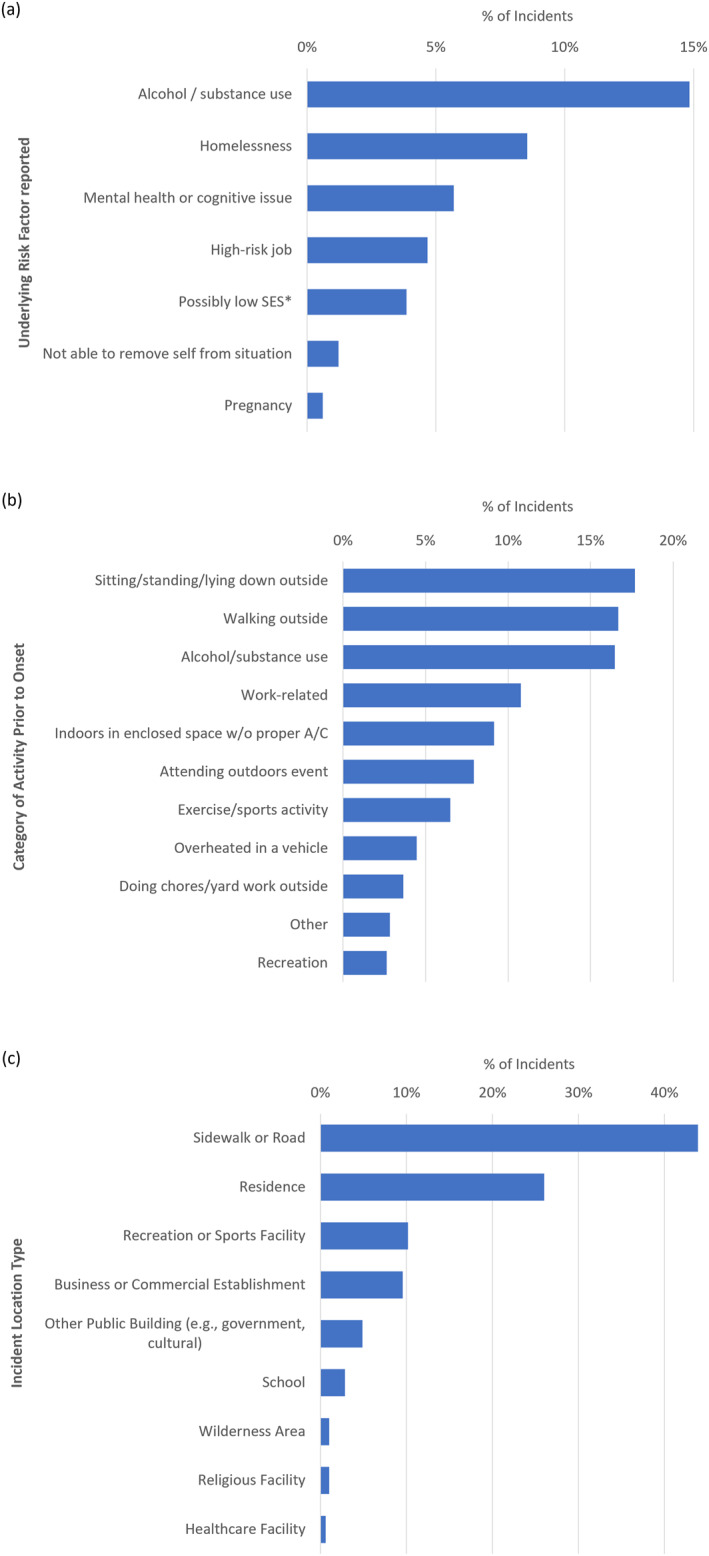
Percent of heat‐related illness EMS incidents (May–September 2016–2022; Richmond, VA) by: (a) underlying risk factor reported, (b) category of activity prior to symptom onset, and (c) incident location type. More than one risk factor was documented for some patients. *Socioeconomic Status (SES).

The majority (63.4%, *n* = 312 out of 492) of heat‐related EMS incidents were either not associated with any underlying risk factors or no risk factors were documented by EMS clinicians (Figure 3a). Alcohol or substance use was reported as an underlying risk factor in 14.8% of cases, while experiencing homelessness was noted among 8.5% of patients, mental health or cognitive issues were reported for 5.7% of patients, and high‐risk occupations were documented for 4.7% of patients. Possibly low socioeconomic status (3.9%), inability to remove oneself from a high‐risk situation (e.g., a child locked in a car) (1.2%), and pregnancy (0.6%) were associated with few cases. Due to previously mentioned limitations of the EMS data set, associated risk factors are likely undercounted in heat‐related EMS incident disparities.

Almost half of all heat‐related EMS incidents during the study period stated the patient was engaged in activities outdoors prior to symptom onset, including sitting, standing, or lying down outside (17.7%), walking outside (16.7%), attending an outdoor event (7.9%), or doing chores or yardwork outside (3.7%) (Figure 3b). Work‐related incidents accounted for 10.8% of cases. Individuals who were indoors in an enclosed space without proper air conditioning accounted for 9.1% of cases, while 4.5% of cases involved a person overheating in a vehicle. Alcohol or substance use was listed as a primary or secondary activity prior to onset in 1 out of every 6 cases (16.5%). In 20.9% of cases, the category of activity prior to onset was unknown. Heat‐related EMS incidents primarily occurred outdoors on a city or residential street, sidewalk, or highway (43.9%) (Figure 3c). Just over a quarter of cases (26.0%) occurred at a residence.

### 3.2 Heat Model Analysis

Air Temperature (AT) observations ranged from 30.3°C to 35.3°C and Heat Index (HI) ranged from 32.3°C to 41.8°C on the day of collection. Land Surface Temperature (LST) values ranged from 24.2°C to 55.2°C. We found positive associations between heat metrics and health emergencies, with hotter areas of the city associated with greater numbers of heat‐related EMS incidents than cooler areas. Mean LST was significantly higher for the 492 incident response locations than for the random set of 492 locations (*t* = 15.08, *p* < 0.01). A similar significant difference was observed between the AT at locations of heat‐related EMS incidents and random locations for AT (*t* = 10.21, *p* < 0.01). Observed heat‐related incident locations were cooler than our random locations for the heat index estimates that include the effects of relative humidity in calculating thermal stress (*t* = −2.49, *p* = 0.99 for one‐tailed test that HI > random).

**Figure 4:**
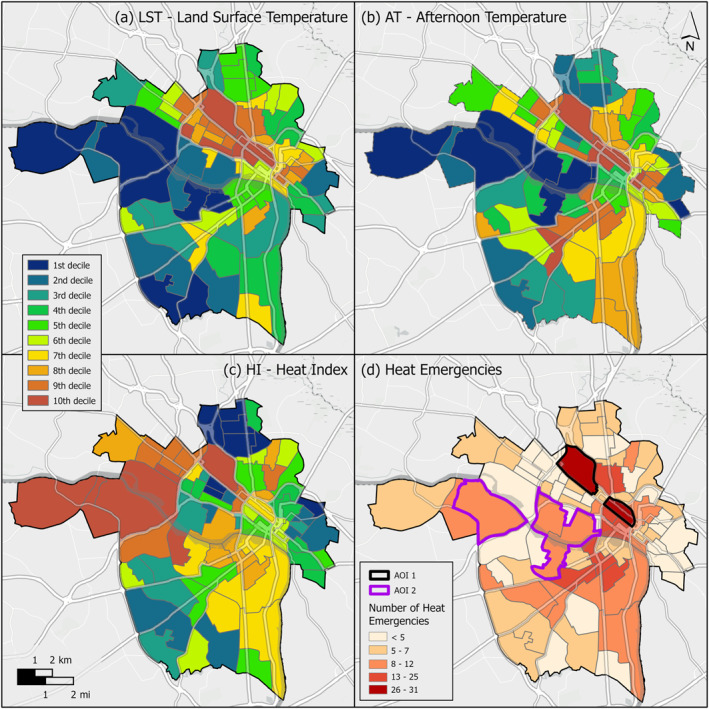
Comparison of heat estimates to heat‐related EMS incidents recorded from 2016 to 2022 for the city of Richmond, Virginia by Census tract. (a) Landsat‐derived land surface temperature, (b) afternoon air temperature based on 15 July 2021 field samples, (c) heat index combining air temperature measurements with relative humidity, (d) the number of reported heat‐related EMS incidents.

At the block‐group scale, 39 of the 190 (20.5%) block groups had no heat‐related EMS incidents, compared to a total of 37 block groups (19.5%) that each experienced 5 or more heat‐related EMS incidents during the study period. The block groups with these large numbers of heat‐related EMS incidents had significantly higher LST (*t* = 2.26, *p* = 0.01) and AT (*t* = 2.45, *p* < 0.01) values than those without any recorded cases. The hottest area of the city, according to both the LST and AT metrics, was also the area of the city with the highest numbers of emergency responses (marked as Area of Interest [AOI] 1 in Figure 4), which included census tracts with as many as 31 and 26 heat‐related EMS incidents. A handful of tracts had eight or more heat‐related EMS incidents and high HI values, but relatively low average land surface and air temperatures (AOI 2 in Figure 4). Overall, there was no significant difference in HI between the 37 block groups with many heat‐related EMS incidents and the 39 block groups with none (*t* = 0.70, *p* = 0.24).

The highest number of heat‐related EMS incidents occurred in the month of July (33.7%) (Figure 1). Heat‐related EMS incidents were highest throughout the afternoon and into the early evening, with 80.5% of incidents occurring between 11:00 a.m. and 7:00 p.m., a peak of 81 incidents during the 3:00 p.m.–4:00 p.m. hr (Figure 1). Nighttime incident counts are relatively low, with only 33 incidents (6.7%) between 9:00 p.m. to 9:00 a.m.

### 3.3 Refuge Analysis

The study area is defined as being confined to Richmond City land (water area excluded) and has an area equal to 156.8 km^2^. Walkshed areas cover 23.9% of the study area (37.5 km^2^). While there were 492 total heat‐related EMS incidents in the Richmond study area, 265 (53.9%) were located within a 1.4 km walkshed (15‐min walk for fast walkers) of an existing heat resilience resource location (e.g., libraries, cooling centers) in Richmond (Table 1). If existing City‐operated community centers were added to these heat resilience resources during a heatwave, the total refuge‐accessible area of the city would increase to 71.3 km^2^ (45.5%) of the city's total area, and 17.3% (85) more heat‐related EMS incidents would have been within these 1.4 km walksheds. A total of 350 (71.1%) heat‐related EMS incidents occurred within this combined refuge walkshed of libraries, cooling centers, and community centers.

**Table 1:** Counts (and Percent of Total Incidents) of Heat‐Related EMS Incidents That Fall Within Library, Cooling Center, and Community Center Refuge Zones

Range	Libraries	Libraries + Cooling centers	Libraries + Cooling centers + Community centers
Slow walking (0.35 km)	25 (5.1%)	45 (9.1%)	75 (15.2%)
Average walking (0.875 km)	100 (20.3%)	130 (26.4%)	212 (43.1%)
Fast walking (1.4 km)	217 (44.1%)	265 (53.9%)	350 (71.1%)

**Figure 5:**
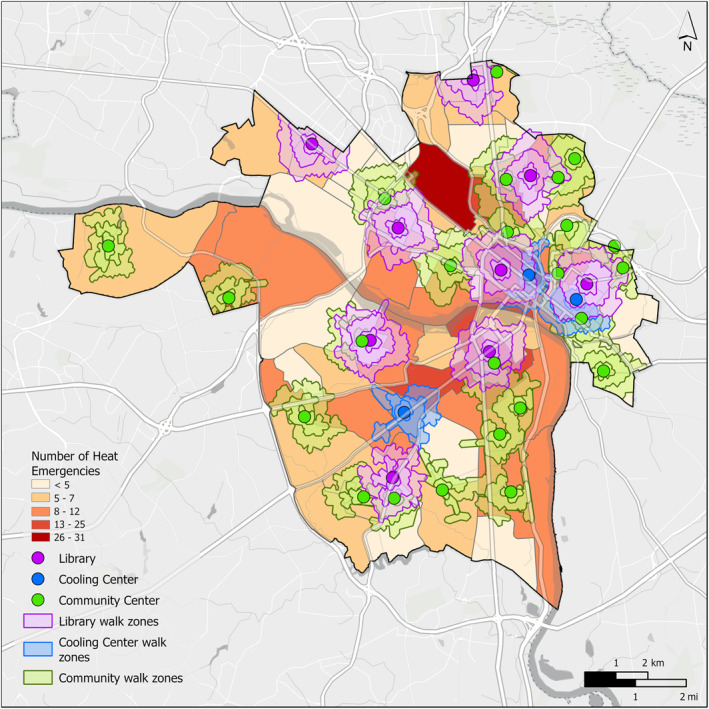
Public refuge, refuge walk zones, and heat‐related EMS incident distributions in Richmond, Virginia.

As the shortest distance between two points, paths traveled straight down the street grid are faster than paths that must make multiple turns, generally forming a diamond‐shaped walk zone (Figure 5). While eight libraries and cooling centers are available in the Northside of Richmond, only four such refuge locations are available in the Southside. Additionally, walk zones in the Southside appear to be smaller in area and more irregular than those in the Northside, potentially indicating lower walkability and/or sidewalk network completeness in the Southside of Richmond. The walksheds of existing cooling centers and libraries across Richmond overlap with the walksheds of 18 community centers, which encompass a high number of heat‐related EMS incidents. Out of the top ten community centers with the most nearby heat‐related EMS incidents, only two are in south Richmond.

### 3.4 Bus Stop Analysis

**Figure 6:**
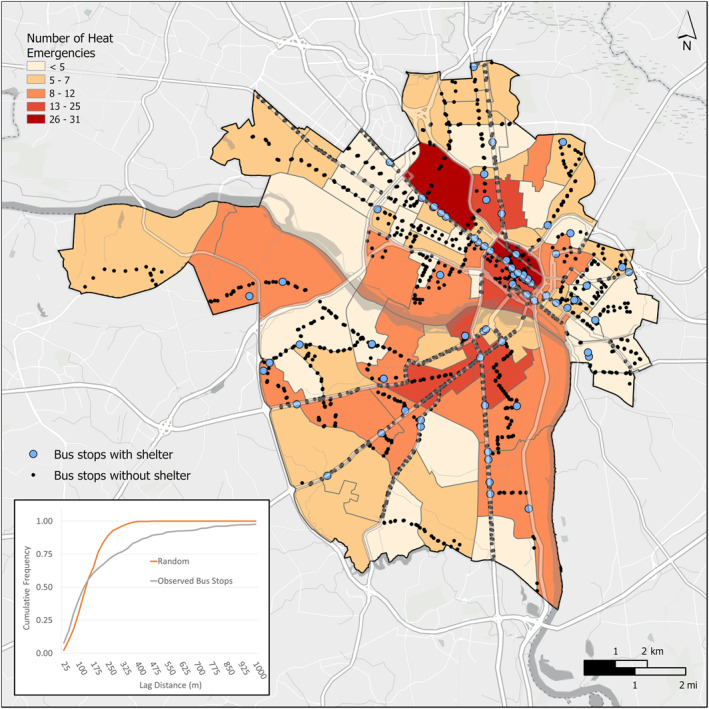
Heat‐related EMS incidents in proximity to GRTC bus stops in Richmond, Virginia. Map inset provides cumulative frequency distribution of the distances from each heat‐related EMS incident to the closest bus stop compared to distances from heat‐related EMS incidents to randomly distributed control points.

The 1,618 bus stops in the city had significantly higher AT than the contrasting set of locations randomly located along Richmond's main and secondary roads (*t* = 16.2, *p* < 0.01). In terms of proximity to heat‐related EMS incidents, although the median values were nearly identical (133 vs. 135 m), the distances from the heat‐related EMS incidents to the bus stops were significantly different than the distances from the heat‐related EMS incidents to the randomly distributed control points according to the Kolmogorov‐Smirnoff test (*D* = 0.20, *p* < 0.01). Notably, there were 50 observed incidents (just over 10% of the total) that occurred at a distance greater than 500 m from any bus stop (Figure 6). At the other extreme, the first two quartiles (50%) of the observed data were located closer to bus stops than expected when compared to the random distribution, and 199 of the incidents (40.4%) occurred at a distance less than 100 m from a bus stop. We did not estimate whether these locations had a shelter at the time of analysis, but only approximately 5% of the network's stops currently have a shaded shelter (Figure 6).

## 4 Discussion

This study aimed to determine whether Richmond's heat‐related EMS incident demographics were aligned with nationally reported heat emergency demographics. Young people (ages 0–19 years) represented a disproportionately low proportion (4.1%) of heat‐related EMS incidents in Richmond, considering this age group makes up 21.2% of the Richmond population (Figure 2a). This may be due to consistent supervision from parents and adults at daycare and out‐of‐school activities during the heat season. At the opposite end of the age spectrum, Richmond appears to be in line with a national study from the CDC that found individuals aged 65 years and older were most susceptible to heat‐related illnesses (Vaidyanathan et al., 2020). Importantly, the proportion (20.7% of cases) of heat‐related EMS incidents among individuals 65 years and older was disproportionately high when compared to Richmond's population makeup, as this age group comprises 13.2% of the Richmond population. According to the CDC study, susceptibility to heat‐related illnesses increases with age largely due to reduced thermoregulatory response caused by preexisting medical conditions (e.g., heart disease, high blood pressure, and diabetes), social isolation, or both factors (Vaidyanathan et al., 2020). More analysis is needed to determine the relationship between heat‐related EMS incidents and rates of chronic conditions which impact cardiovascular regulation across age brackets in Richmond.

Patient race is a significant predictor of heat emergency prevalence in Richmond. Black and African American residents represent a disproportionately large number of heat‐related EMS incidents, while white residents represent a disproportionately small number of heat‐related EMS incidents (Figure 2b). Earlier studies have demonstrated the link between redlining and other historically racist urban planning policies in American cities and environmental inequality (Chakraborty et al., 2023; Hoffman et al., 2020). Black and African American communities are disproportionately impacted by extreme urban heat due to lack of access to resources, including shelter in the form of green spaces and public built infrastructure. Low income and Black and African American households also bear a disproportionately large energy burden, meaning many households are forced to forgo the use of air conditioning due to energy bill and maintenance costs (Maxim & Grubert, 2022).

Geographically, this study is the first to empirically demonstrate that heat‐related EMS incidents in Richmond occur in areas with significantly higher temperatures as measured by land surface and near‐surface air temperatures. These significant correlations indicate that aggregation of heat‐related EMS incidents by census block groups in Richmond is a suitable model for on the ground heat vulnerability compared to afternoon air temperature and land surface temperature. While the aggregation of data for patient privacy purposes does not allow for finer‐scale evaluation of heat emergency clusters, we observe spatial patterns by overlaying the heat and EMS data with information on landscape and land use features which may reveal contributing factors to heat‐related health emergencies.

The apparent heat stress‐cooling effect of low moisture contents in the urban core as described by Chakraborty et al. (2022) did not translate to fewer heat‐related EMS incidents for these areas of Richmond. Although the HI was highest in the western extent of the city when accounting for its relative humidity, this region had relatively few heat‐related EMS incidents during the study period, with the exception of AOI 2. This inverse relationship is likely due to the potentially higher adaptive capacity of more affluent neighborhoods. Affluent areas often have more trees and therefore higher humidity, but the people who live there also might have lower risk due to their ability to escape the heat through air conditioning and other mitigation measures. From these results, it does not appear that humidity‐based heat indices are as good a measure of census block‐scale spatial disparities in heat‐related illness risk for Richmond than the simpler LST and AT metrics that did not account for humidity.

The concentration of heat‐related EMS incidents in AOI 1 may be primarily due to acute exposure to known urban heat islands. AOI 1, encompassing greater Scott's Addition, Jackson Ward, and parts of Downtown Richmond, is a largely built landscape with few urban trees. It is also a major corridor for Richmond bus routes, a destination for cultural, recreational, and musical attractions, and a hub for government and health services relied upon by unhoused individuals. Homelessness was an identified risk factor for incidents in this area (Figure 3a). Activities that preceded symptom onset in the area included walking outside, attending outdoor events or similar outdoor recreational activities (Figure 3b). Yet, AOI 1 contains only 0.07 km^2^ of open park space, covering only 1.5% of the total area.

The correlation of urban heat islands and heat‐related EMS incidents can be explained by the ability of UHI to intensify both acute (sudden and intense) and chronic (prolonged and moderate to intense) exposures, which are both factors of the length of exposure and intensity of exposure (Keith & Meerow, 2022). High vulnerability populations in Richmond include males (Figure 2c), the elderly (Figure 2a) and intoxicated, mentally ill, and/or homeless persons (Figure 3a). The longer a person is exposed to extreme heat without access to shelter, the more likely they are to develop symptoms. Chronic exposures can lead to gradual onset of symptoms, such as dehydration. Even short, acute exposures to extreme heat can result in a medical emergency in vulnerable people who have underlying health concerns (e.g., cardiovascular diseases, asthma) or risk factors (e.g., homelessness, substance use). Clusters of heat‐related EMS incidents in specific geographic regions could also occur due to population density, zoning type, or high traffic areas.

The heat‐related EMS incidents in AOI 2 could be explained by length of exposure and distance from built refuge. AOI 2 encompasses portions of Richmond's nationally ranked James River Park System, which draws millions of visitors annually, as well as Byrd Park, portions of the Northbank Trail and Buttermilk Trail, and the private Willow Oaks Country Club. These open spaces cover 30.6% of AOI 2. The high level of vegetation cover in these spaces and associated high relative humidity can explain the high HI values for AOI 2. These parks and trails are primarily used for outdoor recreation and physical exercise. The isolation from built refuge in these larger parks and duration of use means exposure to extreme heat is more likely. With few access points to trails, users may be 10–15 min or more walking distance from the nearest air‐conditioned building or water fountain at any time.

While the richness of the heat‐related illness data set provides insight into the on‐the‐ground health impact of heat stress in Richmond communities, this data set also presents inherent limitations in the ability to capture all heat‐related illnesses, including certain demographic groups and health disparities. By focusing on EMS incidents, the data set in this paper would miss any heat‐related illness patients who did not interact with the EMS system, such as patients who went directly to an emergency room or who did not receive any medical care for their condition for various reasons (e.g., patients experiencing milder illness or financial inaccessibility). For example, Hispanic or Latino residents represent a disproportionately low number of heat‐related EMS incidents and could be underrepresented in this data set due to several socioeconomic factors, including the cost of medical treatment, access to insurance, or legal status. The result is likely an underreporting rather than over‐reporting of incidents, leading to an underestimation of the true burden of heat‐related morbidity within the community. However, EMS is often the first interaction an individual has with the healthcare system and represents a critical period for early intervention and treatment.

### 4.1 Walkability/Access to Cooling Center Infrastructure

Extreme heat resilience in cities can take on two complementary approaches: management (response to heat threat) and mitigation (physically lowering the temperature of neighborhoods). This study builds on previous results (Allen et al., 2022 and references therein) showing lack of government‐sponsored cooling centers in most heat‐exposed areas, a management‐focused response to urban heat.

Refuge locations in Richmond are currently insufficient to provide free and usefully accessible protection across the most vulnerable areas of the city. While many, if not most, residents have access to sufficient resources to spend time in a private residence or public shop, this is not true for everyone. At current estimates, less than a quarter of the Richmond study area falls within a walkable distance to a refuge zone, even in the high‐traffic downtown areas (Figure 5). The number of cases within the refuge zones grew exponentially as mobility (walking speed) increased, highlighting the necessity of equity in heat resilience planning.

Furthermore, the results of this study identify community centers as essential public infrastructure in the event of heat waves and heat‐related EMS incidents. Most of the benefit of opening community centers occurs at low walking distances (low mobility) (Table 1). In other words, each heat‐related EMS incident that falls within the low (0.35 km) and medium (0.875 km) mobility walksheds surrounding community centers was not already covered by existing refuge zones (Figure 5). However, at a distance of 1.4 km, relatively few additional heat‐related EMS incidents occur in this outer ring. Benefits begin to taper off despite encompassing a larger area. This could be due to clustering of cases around community centers or increasing overlap of coverage between refuge zones. Intentionally sited refuge locations in high use, high heat areas can have the most benefit per location, especially for low‐mobility residents. Southside's only existing cooling center, the Southside Community Services Center on Hull Street Road, exemplifies a well‐sited refuge location, as its walkshed covers a known heat island with high AT and LST in a high‐traffic area (Figures 4 and 5). The irregular shape of this cooling center's walkshed also exemplifies the barrier that poor pedestrian infrastructure poses to accessing refuge in Southside. Furthermore, utilization of cooling centers may be low due to lack of cultural relevance and distrust in City systems.

Additional cooling centers may be needed in areas without coverage. In AOI 1, Scott's Addition lacks access to a cooling center of any kind, while much of the James River Park/Byrd Park area in AOI 2 is too far from built refuge of any kind. As localities identify areas of growth, both in terms of development and public use, built refuge will increasingly become essential heat management infrastructure.

### 4.2 Transit Infrastructure as Heat Resilience

We found that both heat‐related EMS incidents and bus stops were in areas that are significantly hotter than the rest of the city. A majority (79.5%) of heat‐related EMS incidents occurred within only 0.35 km (15‐min walk for slow walking adult) of a bus stop and almost all cases (99.2%) were within 1.4 km (15‐min walk for a fast walker) of a bus stop (Table 1). Bus stops are strategically sited in high‐traffic areas and always along busier roads, which could explain the proximity of heat‐related EMS incidents to bus stops (Figure 6). Bus stops are located close to overheated roadways and, in Richmond, are significantly lacking in shade. Rider safety and comfort can be improved by the construction of benches and shelters at every bus stop. Moreover, the construction of shelters at every bus stop could be viewed as an opportunity to provide built refuge in close proximity to more than ¾ of all heat‐related EMS incidents. Tree planting at bus stops could further cool riders as they wait in the heat. A full network of shelters could provide strategically‐located stops for pedestrians and local residents as they attempt to reach free, public, air‐conditioned refuge locations.

While heat advisories are usually widely televised and shared over the internet, many people are still unaware of high‐risk days for extreme heat exposure (Lane et al., 2014). There are two main information solutions: increase public knowledge of the dangers of heat‐related illnesses and their symptoms, risk factors, and prevention measures; and increase knowledge of publicly available refuge and resources ahead of extreme heat events. Demographics of persons most frequently exposed should be considered when determining outreach strategy, methods, and groups contacted. As males, individuals between the ages of 19–64 years, and members of Richmond's Black and African American community are most likely to experience a heat emergency, particular efforts should be made to identify why these groups are most vulnerable and encourage education and reallocation of resources where necessary. One strategy toward this end is to educate entire communities while focusing on an individual promoter or ambassador to encourage increased community‐wide knowledge (Riley et al., 2012). While there are cooling centers and libraries open to the public in many of the areas of Richmond with heat islands and high numbers of heat‐related EMS incidents, for a variety of reasons (e.g., community knowledge), this refuge is not or cannot always be utilized by the public. Increased free community refuge is not useful unless there is community‐led planning and education involved in the implementation of these programs.

As there is a clear connection between urban heat islands and heat emergencies, policy makers should act expediently to increase free, public refuge facilities to protect urban residents from the already present impacts of climate change on urban life. Historically disenfranchised groups should be brought into planning and decision‐making processes to increase knowledge of heat‐related health illnesses and the precautionary measures that can be taken by individuals and communities to reduce heat‐related illnesses, while simultaneously empowering these groups to utilize these resources. It is clear from the reported activities prior to symptom onset that heat‐related EMS incidents occur while people are going about their everyday lives: walking the dog at the park, sitting in traffic, working in the garden, running errands, or even watching a soccer game. Heat illnesses can impact anyone, including those with stable access to shelter. While most people do not want to make changes to their lifestyle, climate change is increasing the intensity and frequency of extreme heat, which will continue to impact the lives of urban dwellers around the world. As empirical evidence supporting the relationship between climate change and health outcomes grows, cities and their communities should make investments to protect vulnerable residents long into the future.

## Conflict of Interest

The authors declare no conflicts of interest relevant to this study.

## Data Availability

The data used in this study include: 2020 U.S. Census Data and American Community Survey 5‐Year Estimates for Richmond, Virginia (U.S. Census Bureau, 2020); data from the City of Richmond GeoHub for Public Libraries, Community Centers, GRTC bus stops, Richmond City boundary, and James River boundary (Richmond GeoHub, 2020); Landsat Collection 2 Level‐2 Land Surface Temperature (LST) data generated using the thermal infrared band (TIRS) from Landsat 8 imagery of Richmond from 30 June 2021, 11:46 a.m. EDT (USGS, 2021); and ground measurements of ∼2 m ambient air temperature and relative humidity from 15 July 2021 at 3:00–4:00 p.m. EST from CAPA Strategies (CAPA Strategies, 2021). Additionally, a data set of heat‐related EMS responses recorded in the Virginia Pre‐Hospital Information Bridge during the May‐September heat season from 2016–2022 was made available by the Virginia Department of Health Office of Emergency Medical Services under a data use agreement ensuring privacy, data security, and granting VDH OEMS final review of data use and data products. This EMS data is not publicly available as it would compromise individual privacy but may be made available at the discretion of VDH OEMS and the state health commissioner by submitting a data request (VDH OEMS, 2023).
